# Comprehensive molecular pharmacology screening reveals potential new receptor interactions for clinically relevant opioids

**DOI:** 10.1371/journal.pone.0217371

**Published:** 2019-06-06

**Authors:** Keith M. Olson, David I. Duron, Daniel Womer, Ryan Fell, John M. Streicher

**Affiliations:** 1 Department of Pharmacology, College of Medicine, University of Arizona, Tucson, AZ, United States of America; 2 Depomed, Inc., Newark, CA, United States of America; University of North Dakota, UNITED STATES

## Abstract

Most clinically used opioids are thought to induce analgesia through activation of the mu opioid receptor (MOR). However, disparities have been observed between the efficacy of opioids in activating the MOR *in vitro* and in inducing analgesia *in vivo*. In addition, some clinically used opioids do not produce cross-tolerance with each other, and desensitization produced *in vitro* does not match tolerance produced *in vivo*. These disparities suggest that some opioids could be acting through other targets *in vivo*, but this has not been comprehensively tested. We thus screened 9 clinically relevant opioids (buprenorphine, hydrocodone, hydromorphone, morphine, O-desmethyl-tramadol, oxycodone, oxymorphone, tapentadol, tramadol) against 9 pain-related receptor targets (MOR, delta opioid receptor [DOR], kappa opioid receptor [KOR], nociceptin receptor [NOP], cannabinoid receptor type 1 [CB1], sigma-1 receptor [σ1R], and the monoamine transporters [NET/SERT/DAT]) expressed in cells using radioligand binding and functional activity assays. We found several novel interactions, including monoamine transporter activation by buprenorphine and σ1R binding by hydrocodone and tapentadol. Tail flick anti-nociception experiments with CD-1 mice demonstrated that the monoamine transporter inhibitor duloxetine selectively promoted buprenorphine anti-nociception while producing no effects by itself or in combination with the most MOR-selective drug oxymorphone, providing evidence that these novel interactions could be relevant *in vivo*. Our findings provide a comprehensive picture of the receptor interaction profiles of clinically relevant opioids, which has not previously been performed. Our findings also suggest novel receptor interactions for future investigation that could explain some of the disparities observed between opioid performance *in vitro* and *in vivo*.

## Introduction

Opioid drugs interact with the 3 canonical opioid receptors (mu, kappa, delta [MOR, KOR, DOR]) with varying selectivity ratios [1–3]. However, experiments with the MOR knockout (KO) mouse demonstrated that morphine anti-nociception was fully mediated through the MOR, and most clinical opioids are thought to exert their analgesic/anti-nociceptive effects through the MOR [4, 5]. Despite this apparent simplicity, different opioids can show different efficacies and side effect profiles in different patients, leading pharmacologists at times to propose MOR subtypes [6, 7]. In addition, not all opioids produce cross-tolerance; for instance, morphine remains anti-nociceptive at equipotent dosages in oxycodone tolerant and naïve animals [8, 9]. This lack of cross-tolerance is the basis behind clinical opioid rotation to retain treatment potency and efficacy [10, 11]. These disparities suggest that something beyond simple activation of the MOR is responsible for the different effects of clinical opioids.

Further supporting this hypothesis, differences between the performance of clinical opioids in simplified MOR-expressing cell systems and in promoting anti-nociception *in vivo* have been observed. The operational efficacy of a drug, τ, is the ability of an agonist to produce a response and the efficiency by which it produces that response in the receptor system, and can be measured both *in vitro* and *in vivo* [12]. Comparing *in vitro* and *in vivo* τ values of clinical opioids has shown that several clinical opioids behave differently between the systems. For instance, morphine, hydromorphone, and oxycodone have low τ values *in vitro* (3.3–5.2), but much higher τ values *in vivo* (20–39), suggesting that the drugs are much more efficient and efficacious *in vivo* than simple receptor activation *in vitro* would suggest. Similarly, methadone and morphine have the same τ value of 39 *in vivo*, but methadone has a much higher τ value *in vitro* (18.2 vs. 5)[13–15]. Disparities have also been observed between *in vitro* desensitization and *in vivo* tolerance. Higher efficacy generally correlates with increased receptor desensitization *in vitro* [16]; however, the high efficacy agonist etorphine produces less anti-nociceptive tolerance *in vivo* than the lower efficacy oxycodone or hydrocodone [15].

One possibility to explain these disparities is that clinical opioids could be interacting with targets apart from the MOR to produce their different effects. Some drugs are well established as interacting with multiple receptor systems; buprenorphine has been shown to act as a KOR antagonist and nociception receptor (NOP) agonist [17, 18], and limited evidence suggests that oxycodone can promote anti-nociception through the DOR and/or KOR [19]. Tapentadol and tramadol are also established as MOR agonists and target one or more of the monoamine transporters (norepinephrine [NET], serotonin [SERT], and dopamine [DAT]) as inhibitors [20, 21]. Several opioids, particularly morphine, have been shown to interact with Toll-Like Receptors, which could help drive neuroinflammation and other side effects caused by chronic opioid treatment [22]. However, in general, clinically relevant opioids have not been systematically screened or tested for interactions with atypical non-opioid receptor targets.

We thus performed a molecular pharmacology screen of 9 clinically relevant opioids (buprenorphine, hydrocodone, hydromorphone, morphine, tramadol, O-desmethyl-tramadol [the active metabolite of tramadol], oxycodone, oxymorphone, and tapentadol) tested against 9 pain-related receptor targets expressed in non-neuronal cell models. These targets were the MOR, DOR, KOR, NOP, cannabinoid receptor type 1 (CB1), sigma-1 receptor (σ1R), and the NET, SERT, and DAT (**Table 1**). DOR, KOR, and NOP agonists all can produce anti-nociception, and are known to interact with some clinical opioids [23–25]. CB1 agonists like Δ9-tetrahydrocannabinol can also produce anti-nociception, and the CB1 and MOR systems extensively interact in the brain [26]. The σ1R is an intracellular chaperone-like protein, that has been shown to have a role in pain and anti-nociception, and has been shown to interact with a few non-clinical opioids like SKF-10047 [27, 28]. The inhibition of NET and SERT has been shown to be responsible in part for the anti-nociceptive efficacy of tapentadol and tramadol as above [20, 21], and the DAT inhibitor bupropion has been used to treat neuropathic pain [29]. We tested the opioids against these targets using radioligand binding to assess drug binding and affinity, and functional assays to assess potency and efficacy. We found several novel interactions, suggesting future directions for investigation that may explain the disparities observed between clinical opioids *in vitro* and *in vivo*.

**Table 1:** Screened clinical opioids and receptor targets.

Targets	Drugs
Mu Opioid Receptor (MOR)	Buprenorphine
Delta Opioid Receptor (DOR)	Hydrocodone
Kappa Opioid Receptor (KOR)	Hydromorphone
Nociceptin Receptor (NOP)	Morphine
Cannabinoid Receptor Type 1 (CB1)	O-desmethyl-tramadol
Sigma-1 Receptor (σ1R)	Oxycodone
Norepinephrine Transporter (NET)	Oxymorphone
Serotonin Transporter (SERT)	Tapentadol
Dopamine Transporter (DAT)	Tramadol

## Materials and methods

### Cell lines and cell culture

Chinese Hamster Ovary (CHO) cells stably expressing the human MOR (#ES-542-C), KOR (#ES-541-C), NOP (#ES-230-C), and CB1 (#ES-110-C) were all obtained from PerkinElmer (Waltham, MA). The human DOR-CHO cell line expressing a 3X-hemagglutinin (HA) N-terminal tag was previously created and characterized in our lab [30]. For the σ1R, NET, SERT, and DAT, stable expression vectors were obtained from Genecopoeia (Rockville, MD), all human with an N-terminal intracellular HA tag, contained in a pEZ-M06 vector. Each expression construct was electroporated into Human Embryonic Kidney (HEK293, #CRL-1573, American Type Culture Collection, Manassas, VA) cells, and selected with 500 μg/mL of G418. Selected populations were used for experiments after analysis for expression by immunocytochemistry and saturation radioligand binding (see below).

All cells were maintained in a 37°C, 5% CO_2_ humidified incubator. All CHO cells were grown in 1:1 DMEM/F12 medium, with 10% heat-inactivated Fetal Bovine Serum (FBS, #10437028, Thermo Fisher Scientific, Waltham, MA) and 1X penicillin-streptomycin (P/S) supplement. All HEK293 cells were grown in MEM medium, with 10% heat-inactivated FBS (#10437028, Thermo Fisher Scientific) and 1X P/S. The SERT-HEK cells were grown using dialyzed FBS (#26-400-036, Fisher Scientific, Hampton, NH) to remove endogenous serotonin. The KOR-CHO propagation cultures were further maintained with 5 μg/mL blasticidin, while all other propagation cultures were maintained in 500 μg/mL G418. All experiments were carried out within 20 passages of the founding stock. The cells were monitored for mycoplasma contamination by DAPI staining and confocal microscopy, and all cells used in this project were mycoplasma negative.

Each independent experiment for radioligand binding and ^35^S-GTPγS coupling consisted of 3 x 15cm culture dishes grown to confluency (no G418 or blasticidin). The cells were collected with 5 mM EDTA in Dulbecco’s Phosphate-Buffered Saline (dPBS) without calcium or magnesium, and centrifuged at 1,000 rpm for 5 minutes. The supernatant was aspirated, and the resulting cell pellet stored at -80°C until needed.

### Materials

^3^H-Diprenorphine (#NET1121250UC), ^3^H-Nociceptin (#NET1130050UC), ^3^H-CP55,940 (#NET1051025UC), ^3^H-Mazindol (#NET816250UC), ^3^H-DTG (#NET986250UC), and ^35^S-GTPγS (#NEG030H250UC) were all obtained from PerkinElmer. Guanosine diphosphate (GDP) was obtained from Sigma Aldrich (St. Louis, MO), stored at -20°C under desiccation, made fresh for each experiment, and discarded after 60 days. All clinical opioid drugs were obtained from Depomed, Inc. (Newark, CA; authors on this manuscript). Naloxone, nociceptin, BD1008, S-duloxetine, U50,488, and GBR12909 were obtained from Fisher Scientific (distributed from Tocris, R&D). WIN55,212 was obtained from Fisher Scientific (distributed from Cayman Chemical). Endomorphin-2 and DPDPE were synthesized using a standard solid phase peptide synthesis protocol, and validated for purity (>95%) by HPLC and identity by mass spectrometry. All compound powders were stored as recommended by the manufacturer. 10 mM drug stock solutions were made in vehicle and stored at -20°C for no more than 30 days. Standard chemicals and buffers were purchased from Fisher Scientific with a minimum purity of 95%.

### Radioligand binding

The basic protocol for our binding experiments is reported in [30–32]. We optimized the binding buffer, time, and temperature for each cell line using the appropriate radioligand, these conditions are reported in **Table 2**. ^3^H-Diprenorphine was used for all 3 opioid cell lines and ^3^H-Mazindol for all 3 transporter cell lines as those ligands are non-selective between the subtypes. For saturation binding, increasing concentrations of radioligand was used, for competition binding, a fixed concentration. Radioligand was combined with 20–40 μg of membrane protein, and for competition binding, a concentration curve of competitor opioid or positive control ligand, to a 200 μL volume in round bottom polypropylene 96 well plates. Non-specific binding (0%) was determined in the presence of 10 μM of positive control ligand, and subtracted from maximum binding to calculate the specific binding (100%). The reactions were incubated at the time and temperature noted in **Table 2**, and terminated by rapid filtration through 96 well GF/B filter plates (PerkinElmer) using a 96 well format Brandel (Gaithersburg, MD) cell harvester. The plates were dried, 40 μL of Microscint PS (PerkinElmer) was added, and the data was collected using a 96 well format 6 detector MicroBeta2 scintillation counter (PerkinElmer). The resulting data was normalized as above. For saturation binding, the K_D_ and B_MAX_ were calculated using a 1 site specific binding fit using Prism 7.02 (GraphPad, La Jolla, CA). For competition binding, the K_I_ was calculated using the previously measured K_D_ of each radioligand in that specific cell line using a 1 site competition binding fit from Prism 7.02 (GraphPad). The resulting data was calculated separately for N ≥ 3 independent experiments performed at least in duplicate, and reported as the mean ± SEM.

**Table 2:** Optimized conditions for radioligand binding and functional assays.

Cell Line	Binding Buffer	Radioligand	Time & Temp.	Activity Assay Buffer
MOR-CHO	50 mM Tris-HCl pH 7.4, 1 mM PMSF	^3^H-Diprenorphine	1 hr, 30°C	50 mM Tris-HCl pH 7.4, 100 mM NaCl, 5 mM MgCl_2_, 1 mM EDTA, 1 mM PMSF, 40 μM GDP
DOR-CHO				
KOR-CHO				
NOP-CHO		^3^H-Nociceptin		
CB1-CHO		^3^H-CP55,940		20 mM HEPES pH 7.15, 200 mM NaCl, 3 mM MgCl_2_, 15 μM GDP
DAT-HEK	50 mM HEPES pH 7.15, 125 mM NaCl, 3.3 mM EDTA, 0.1% ascorbic acid	^3^H-Mazindol	1.5 hrs, 37°C	50 mM HEPES in Hank’s Balanced Salt Solution (HBSS; no Calcium or Magnesium)
NET-HEK	50 mM HEPES pH 7.15, 125 mM NaCl, 3.3 mM EDTA, 5 mM KCl, 1X Millipore Peptidase Inhibitor			
SERT-HEK				
σ1R-HEK	50 mM Tris, pH 8.0	^3^H-DTG	4 hrs, 37°C	N/A

### ^35^S-GTPγS coupling

Our protocol for ^35^S-GTPγS coupling is also reported in [30–32]. Concentration curves of opioid or positive control drugs were combined with 10–15 μg of membrane protein and 0.1 nM ^35^S-GTPγS (PerkinElmer) at a 200 μL volume as above using the assay buffer reported in **Table 2**. The reactions were incubated at 30°C for 1 hour. For the KOR antagonist experiments, concentration curves of potential antagonists were incubated with the membrane protein for 5 minutes prior to adding the ^35^S-GTPγS and a 100 nM fixed concentration of U50,488, which was then incubated for 1 hour at 30°C. Reactions were collected and measured as for the radioligand binding. The resulting data was normalized to the stimulation caused by positive control agonist (100%) and vehicle (0%). The data was then fit with a 3 variable agonist or antagonist curve, providing the potency (EC/IC_50_) and efficacy (E/I_MAX_), using Prism 7.02 (GraphPad). The resulting data was calculated separately for N ≥ 3 independent experiments performed at least in duplicate, and reported as the mean ± SEM.

### Monoamine transporter activity assay

A monoamine transporter uptake assay kit (#R8173) from Molecular Devices (San Jose, CA) was used to measure transporter activity in all 3 monoamine transporter cell lines. The manufacturer’s protocol was followed using the buffer reported in **Table 2**. 96 well clear bottom black walled plates were coated with bovine collagen type I, and plated with 60,000–80,000 cells per well in growth medium with recovery overnight. Concentration curves of opioids or positive control ligands were incubated with the cells for 20 minutes. The transporter dye was then added and equilibrated for 45 minutes at 37°C. The plates were then read on a BioTek Synergy plate reader (Winooski, VT) with 485 nm excitation and 528 nm emission filters. The resulting data was fit using 3-variable non-linear curve regression using Prism 7.02 (GraphPad). Potency (IC_50_) values were fit directly, and efficacy (I_MAX_) values were calculated by comparison to the positive control inhibitor (100%). The resulting data was calculated separately for N ≥ 3 independent experiments performed at least in duplicate, and reported as the mean ± SEM.

### Animals

Male CD-1 (a.k.a. ICR) mice in age-matched cohorts of 4–8 weeks of age were obtained from Charles River Laboratories (Wilmington, MA). The mice were allowed to recover for at least 5 days after shipment prior to experimentation, and no more than 5 mice were housed in 1 cage. The mice were maintained in an AAALAC-accredited vivarium at the University of Arizona on 12 hour light-dark cycles in temperature and humidity controlled rooms with standard chow and water available *ad libitum*, including during experiments. All procedures were in accordance with the NIH Guide for the Care and Use of Laboratory Animals, and all procedures were approved by the University of Arizona IACUC.

### Tail flick anti-nociception assay

The mice were randomized into treatment groups, and the experimenter was blinded to treatment group by the delivery of coded drug vials. The code was revealed after the end of the experiment. Our tail flick assay protocol is reported in [32, 33]. The baseline to withdraw the tail from 52°C water was recorded using a stopwatch, with a 10 second cutoff to prevent tissue damage. Baseline measurements were recorded, followed by intraperitoneal injection of duloxetine (20 mg/kg) or vehicle (1% Tween80 in saline) for 10 minutes. Subcutaneous equi-efficacious ~A_50_ doses of buprenorphine (0.2 mg/kg), oxymorphone (0.3 mg/kg) or vehicle (saline) was then injected, and tail flick latencies recorded over a 2 hour time course. The resulting raw data in seconds was reported as the mean ± SEM, and analyzed using a 2 Way ANOVA with Fisher’s Least Significant Difference post-hoc test. Significance was set at *p* < 0.05. Area under the curve (AUC) analysis was also performed using Prism 7.02 (GraphPad).

## Results

### Clinical opioid screening by competition radioligand binding

The K_D_ and B_MAX_ values for our MOR, DOR, and KOR cells determined using saturation radioligand binding with ^3^H-Diprenorphine were reported in [30, 31]. The K_D_ and B_MAX_ values for our remaining cell lines were measured using the radioligands noted in **Table 2** and are reported in **Table 3**. The NOP-CHO and CB1-CHO lines are commercial, however, the σ1R and monoamine transporter lines were created during this project, so this analysis validates the successful creation of those lines. All radioligands used (**Table 2**) are well-established ligands for their target, with ^3^H-Diprenorphine non-selective between the opioid receptors and ^3^H-Mazindol non-selective between the monoamine transporters. The resulting K_D_ values are in the expected range for the ligands used based on validated data provided by PerkinElmer, and the B_MAX_ values demonstrate high receptor expression in all cell lines (≥0.75 pmol/mg).

**Table 3:** Cell line saturation binding results.

	MOR-CHO	DOR-CHO	KOR-CHO	NOP-CHO	CB1-CHO	σ1R-HEK	SERT-HEK	NET-HEK	DAT-HEK
**K**_**D**_ **(nM)**	5.2 ± 2.4	1.7 ± 0.2	1.2 ± 0.1	0.075 ± 0.01	0.92 ± 0.1	13 ± 1.1	16 ± 1.9	7.8 ± 2.1	23 ± 4.8
**B**_**MAX**_ **(pmol/mg)**	9.3 ± 3.7	0.62 ± 0.08	2.1 ± 0.3	0.75 ± 0.02	9.1 ± 0.5	10 ± 0.4	1.7 ± 0.1	1.3 ± 0.1	2.0 ± 0.3

Saturation radioligand binding was performed for the listed cell lines as described in the Materials and Methods. The binding constant (K_D_) and receptor expression (B_MAX_) are reported as the mean ± SEM from N = 3 independent experiments.

**Fig 1:**
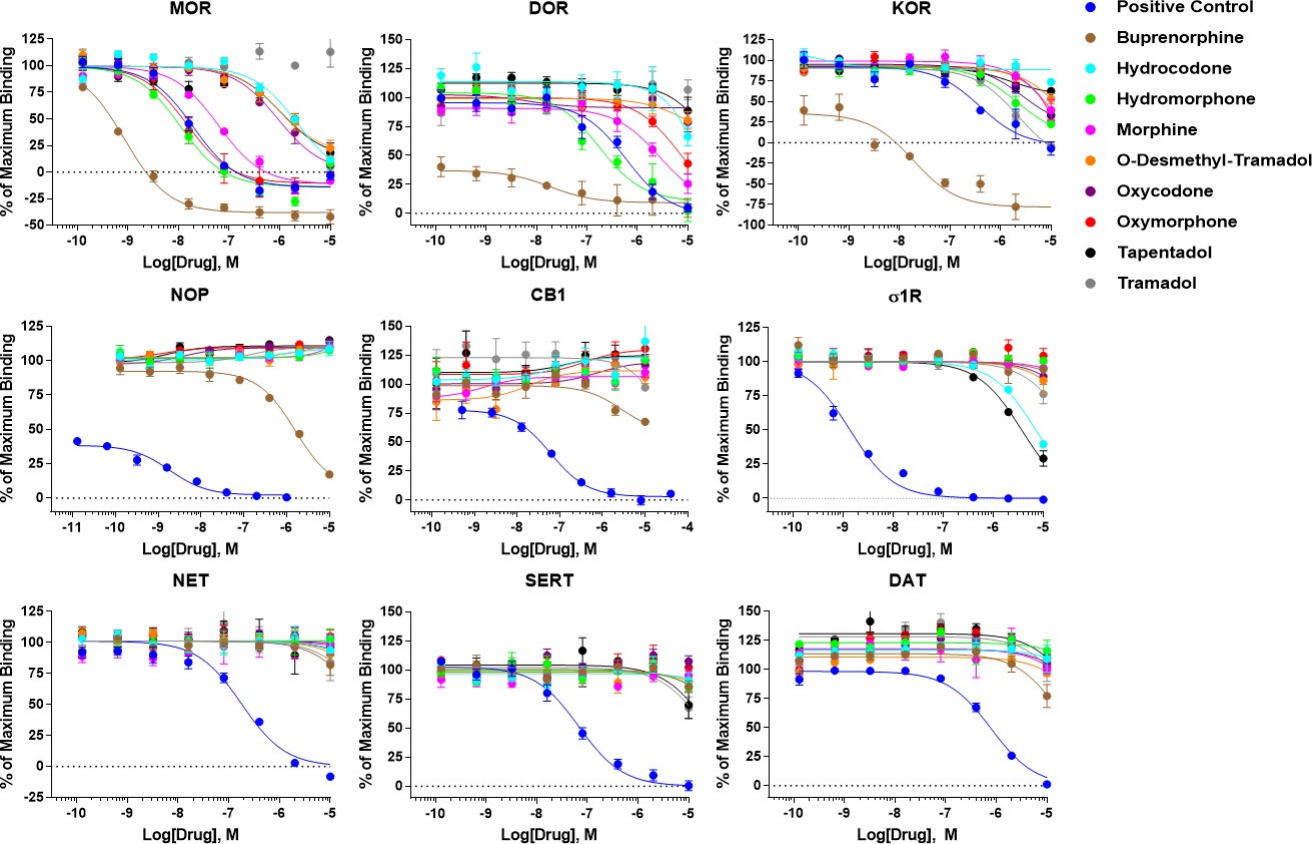
Summary concentration-response curves for radioligand binding. Competition radioligand binding was performed as described in the Materials and Methods for all 9 clinical opioids and a positive control competitor ligand at all 9 receptor targets. Positive control: MOR/DOR/KOR = naloxone, NOP = nociceptin, CB1 = WIN55,212, σ1R = BD1008, NET/SERT/DAT = S-duloxetine. The curves shown here are reported as the mean ± SEM of the mean values calculated separately from N ≥ 3 independent experiments performed in duplicate. The data is further reported as the % of maximum binding, which is determined from only radioligand present and no competitor (100%) and as the non-specific binding, which is radioligand in the presence of 10 μM positive control compound (0%).

Competition radioligand binding was then performed using a fixed concentration of radioligand and concentration curves of opioids or positive control competitor ligand. Summary curves for each drug against each receptor target are shown in **Fig 1**. Each individual curve from N ≥ 3 independent experiments was used to separately calculate the binding affinity, K_I_, using the previously established K_D_ values of each radioligand at each receptor. These K_I_ values are reported as the mean ± SEM in **Table 4**.

**Table 4:** Competition radioligand binding affinity values.

Drug	Target Binding Affinity Determined by Competition Radioligand Binding–K_I_ (nM)								
	MOR	DOR	KOR	NOP	CB1	σ1R	NET	SERT	DAT
**Buprenorphine**	0.90 ± 0.1	34 ± 27	27 ± 13	430 ± 100	>2000	NC	NC	NC	>2000
**Hydrocodone**	1800 ± 470	>2000	NC	NC	NC	4000 ± 1300	NC	NC	NC
**Hydromorphone**	9.4 ± 2.6	310 ± 150	1600 ± 720	NC	NC	NC	NC	NC	NC
**Morphine**	74 ± 18	2500 ± 720	>2000	NC	NC	NC	NC	NC	NC
**O-Desmethyl-Tramadol**	1300 ± 290	NC	>2000	NC	NC	NC	NC	NC	NC
**Oxycodone**	780 ± 170	NC	>2000	NC	NC	NC	NC	NC	NC
**Oxymorphone**	11 ± 1.8	>2000	>2000	NC	NC	NC	NC	NC	NC
**Tapentadol**	2100 ± 84	NC	>2000	NC	NC	2600 ± 410	NC	>2000	>2000
**Tramadol**	NC	NC	890 ± 33	NC	NC	NC	NC	>2000	>2000
**Naloxone**	14 ± 1.9	520 ± 110	270 ± 46						
**Nociceptin**				0.71 ± 0.3					
**WIN55,212**					33 ± 4.8				
**BD1008**						0.81 ± 0.3			
**S-Duloxetine**							110 ± 9.7	51 ± 10	520 ± 68

The curves from **Fig 1** were used to calculate the binding affinity (K_I_) of each drug at each target using the previously measured K_D_ of each radioligand at each cell line (see Materials and Methods). The K_I_ values are reported as the mean ± SEM calculated separately from N ≥ 3 independent experiments. NC = not converged (no binding detected). >2000 = incomplete curve without full ligand displacement at 10 μM, suggesting a K_I_ > 2,000 nM.

Each positive control compound (naloxone for MOR/DOR/KOR; Nociceptin for NOP; WIN55,212 for CB1; BD1008 for σ1R; S-duloxetine for NET/SERT/DAT) returned K_I_ values in the expected range for each target, validating each assay (e.g. [3]). Each clinical opioid, with the exception of tramadol, bound to the MOR, also as expected. As O-desmethyl-tramadol is considered the MOR-active metabolite of tramadol, this is expected [21], and tramadol has been reported to have a very low K_I_ of >10 μM at the MOR [3]. The rank order of MOR binding affinities also closely matched the rank order of affinities expected from the literature (buprenorphine > hydromorphone = oxymorphone > morphine > oxycodone > O-desmethyl-tramadol > hydrocodone > tapentadol > tramadol)[3]. Most compounds also showed less affinity for the DOR and KOR, with some losing binding to these receptors entirely, with the exception of tramadol, which demonstrated KOR binding but no MOR or DOR binding.

For the NOP, only buprenorphine showed binding, which is expected as buprenorphine is a NOP partial agonist [34]. Interestingly, buprenorphine was the only clinical opioid to show weak binding to the CB1, which was unexpected. The σ1R also showed unexpected interactions, in that hydrocodone bound to the σ1R at only 2.3 fold vs. MOR affinity and tapentadol bound to the σ1R at nearly the same affinity as the MOR. For the transporters, buprenorphine showed unexpected weak binding to the DAT, while tapentadol and tramadol showed expected binding to SERT with unexpected binding to DAT [20, 21]. No clinical opioid binding was detected for the NET, which was not expected for tramadol and tapentadol. Overall these results demonstrated many expected interactions, as well as potential unexpected binding partners, particularly the σ1R. It is important to note that there is no established high-throughput signaling assay for the σ1R, so most drug discovery groups screen against this target by radioligand binding alone, as we have done here [35, 36].

### Clinical opioid functional activity

Radioligand binding is a useful tool to demonstrate ligand binding to a target, but reveals no information about compound functional activity. We thus followed our binding screen with functional assays to reveal ligand potency and efficacy at each target. The MOR, DOR, KOR, NOP, and CB1 receptors are all Gα_I_-coupled G protein coupled receptors (GPCRs), which are well-suited to the ^35^S-GTPγS coupling assay [30–32]. On the other hand, the NET, SERT, and DAT are all transporters instead of GPCRs, for which the ^35^S-GTPγS assay would not work. We thus utilized a monoamine transporter uptake kit from Molecular Devices (see Materials and Methods) to measure ligand activity at these targets.

**Fig 2:**
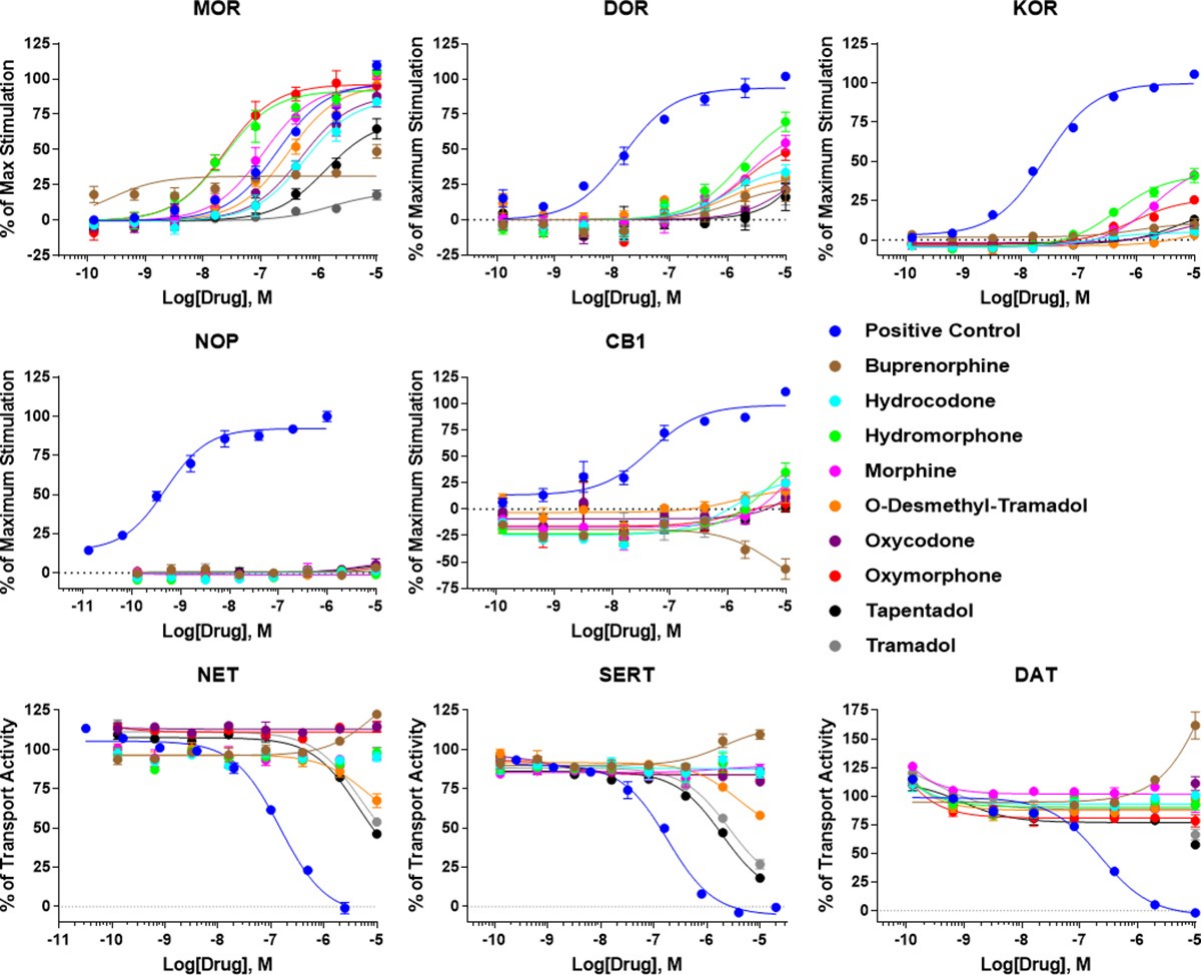
Summary concentration-response curves for receptor functional assays. ^35^S-GTPγS coupling was performed for the MOR, DOR, KOR, NOP, and CB1 in agonist mode and a transport uptake assay was performed for NET, SERT, and DAT, all as described in the Materials and Methods. All 9 clinical opioids and a positive control agonist or transport inhibitor was tested against 8 receptor targets (minus the σ1R). Positive control: MOR = endomorphin-2, DOR = DPDPE, KOR = U50,488, NOP = nociceptin, CB1 = WIN55,212, NET/DAT = S-duloxetine, SERT = GBR12909. The data was normalized to the stimulation caused by positive control compound (100%) and vehicle (0%). The data was further reported as the mean ± SEM of the mean values calculated independently from N ≥ 3 independent experiments performed in duplicate.

Each clinical opioid and a positive control compound (agonist, antagonist, or transport inhibitor as appropriate) was tested against each receptor target (except the σ1R) using these functional assays. The summary curves for each drug and target are shown in **Fig 2**. It is important to note that the ^35^S-GTPγS assay performed in this mode in **Fig 2** will only show agonist activity as positive deflections on the Y axis, while antagonists would show no effect. The transporter assay by contrast can show transport activation or inhibition as positive or negative deflections on the Y axis, respectively. Each individual curve from N ≥ 3 independent experiments was used to separately calculate the potency (EC/IC_50_) and efficacy (E/I_MAX_) of each drug at each target, and the efficacy was calculated by normalization to the maximum effect caused by the positive control ligand (100%). These values are reported as the mean ± SEM in **Table 5**. Each positive control compound showed expected potency values and high efficacy, validating the assays.

**Table 5:** Functional assay potency and efficacy values.

Drug	Target Functional Activity by ^35^S-GTPγS or Transporter Assay															
	MOR		DOR		KOR		NOP		CB1		NET		SERT		DAT	
	EC_50_ (nM)	E_MAX_ (%)	EC_50_ (nM)	E_MAX_ (%)	EC_50_ (nM)	E_MAX_ (%)	EC_50_ (nM)	E_MAX_ (%)	EC_50_ (nM)	E_MAX_ (%)	IC_50_ (nM)	I_MAX_ (%)	IC_50_ (nM)	I_MAX_ (%)	IC_50_ (nM)	I_MAX_ (%)
**Buprenorphine**	<0.1	35 ± 4	1700 ± 520	25 ± 9	1100 ± 310	9.6 ± 2.9	NC	NC	>2000	(-38)	>2000	(-26)	>2000	(-20)	>2000	(-67)
**Hydrocodone**	470 ± 52	87 ± 2	1400 ± 560	42 ± 7	240 ± 130	9.8 ± 3.6	NC	NC	1400 ± 690	54 ± 6	NC	NC	NC	NC	NC	NC
**Hydromorphone**	39 ± 22	94 ± 1	1900 ± 430	83 ± 9	460 ± 100	46 ± 5	NC	NC	>2000	(59)	NC	NC	NC	NC	NC	NC
**Morphine**	130 ± 47	96 ± 4	2200 ± 290	68 ± 8	>2000	(55)	NC	NC	>2000	(42)	NC	NC	NC	NC	NC	NC
**O-Desmethyl-Tramadol**	360 ± 110	97 ± 2	360 ± 240	38 ± 14	>2000	(5.9)	NC	NC	NC	NC	>2000	(29)	>2000	(34)	NC	NC
**Oxycodone**	460 ± 100	90 ± 8	NC	NC	>2000	(9.6)	NC	NC	NC	NC	NC	NC	NC	NC	NC	NC
**Oxymorphone**	23 ± 5	98 ± 6	2000 ± 500	60 ± 24	970 ± 190	29 ± 2	NC	NC	NC	NC	NC	NC	NC	NC	NC	NC
**Tapentadol**	1300 ± 530	73 ± 7	NC	NC	>2000	(13)	NC	NC	NC	NC	>2000	(61)	>2000	(68)	>10,000	(19)
**Tramadol**	3100±2100	26 ± 6	NC	NC	NC	NC	NC	NC	NC	NC	>2000	(57)	>2000	(62)	>10,000	(22)
**Endomorphin-2**	200 ± 32	97 ± 2														
**DPDPE**			17 ± 4	99 ± 3												
**U50,488**					16 ± 4	99 ± 2										
**Nociceptin**							0.26 ± 0.03	100 ± 0.2								
**WIN55,212**									52 ± 15	92 ± 3						
**S-Duloxetine**											140 ± 9	100 ± 0.2			210 ± 14	100 ± 5
**GBR12909**													180 ± 12	100 ± 0.4		

The curves from **Fig 2** were used to calculate the potency (EC/IC_50_) and efficacy (E/I_MAX_) values for each drug at each receptor target. The efficacy was calculated compared to the max stimulation or transport inhibition caused by the positive control compound (100%) or vehicle (0%). The values are reported as the mean ± SEM from N ≥ 3 independent experiments. NC = not converged (no functional activity detected). <0.1 = incomplete curve on the low concentration end but max efficacy suggesting a potency <0.1 nM. >2000/>10,000 = incomplete curve on the high concentration end, preventing accurate determination of potency, which is likely >2000/10,000 nM. () = for incomplete curves, the maximum efficacy at 10 μM is reported. Negative E/I_MAX_ = negative efficacy when compared to the positive control compound; for CB1 this means inverse agonism and for NET/SERT/DAT this means transporter activation.

Our results in general appear to be more sensitive in the activity assays vs. binding assay, with some compounds showing detectable activity but no detectable binding affinity. For instance, tramadol shows no detectable binding at the MOR, but does show low potency but detectable partial agonist activity. The potential reasons for this are explored in the Discussion section. The compounds show expected agonist activity at the MOR, with potency values similar to the binding affinities reported in **Table 4** and the same rank order of compound potencies [3]. Buprenorphine (34.7%), tapentadol (73.0%) and tramadol (25.5%) act as partial agonists, while the other compounds act as full agonists (≥87.1%), also as expected. These MOR findings further validate our results as they are consistent with previously published studies (e.g. [3]).

At the DOR, all compounds except oxycodone, tapentadol, and tramadol showed moderate to low potency agonist activity. Notably, O-desmethyl-tramadol displayed no DOR binding, but moderate potency DOR agonist activity (361 nM). Buprenorphine (34 to 1684 nM) and hydromorphone (306 to 1860 nM) show marked shifts to lower potency from binding to function, potentially demonstrating low intrinsic efficacy for these compounds at the DOR; the remaining compounds show approximately the same affinity and potency. With the exception of hydromorphone (83.0%), all active opioids at the DOR display partial agonist activity (24.8–67.8%)(**Table 5**).

**Fig 3:**
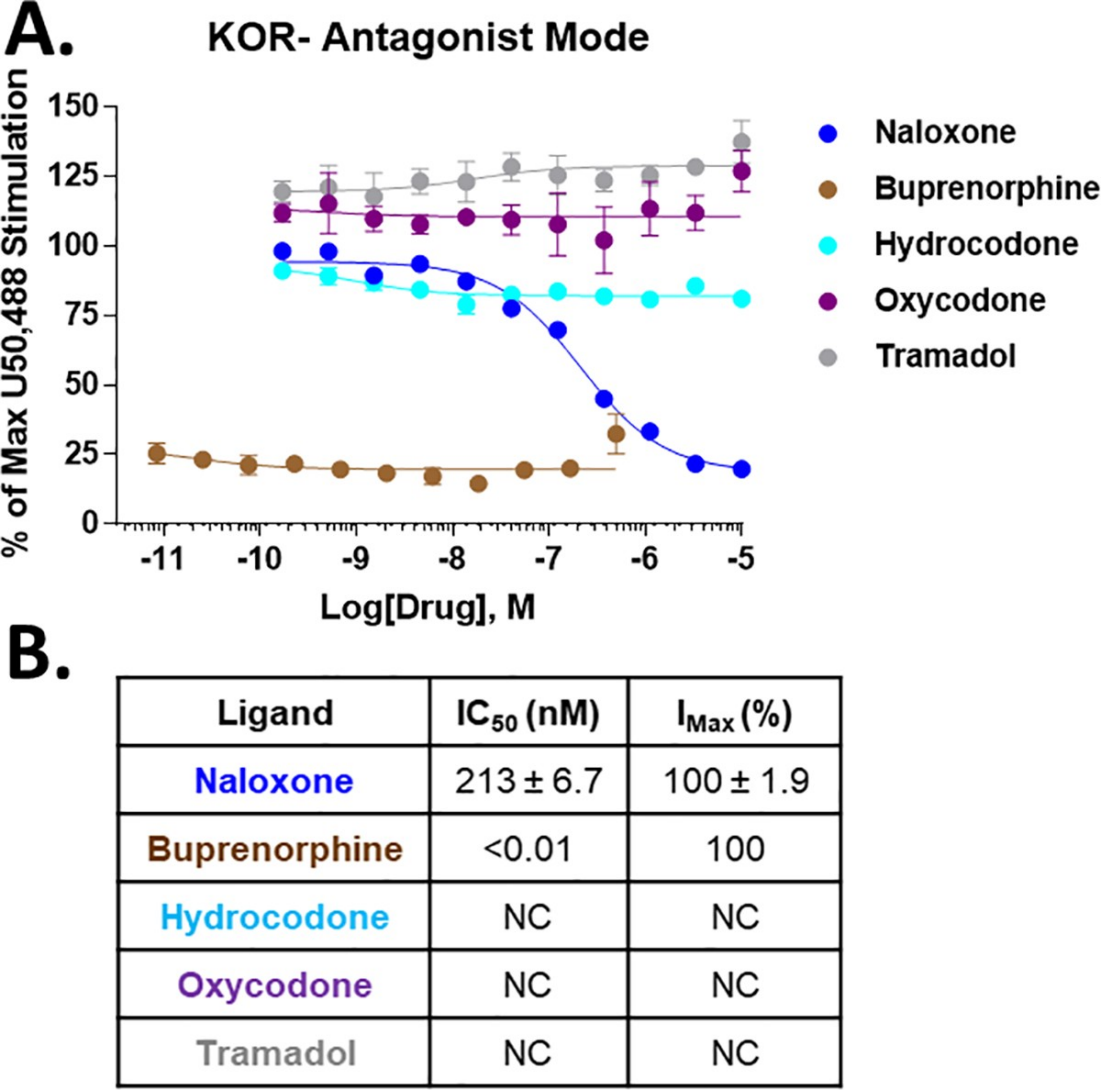
Selected opioid compounds tested as KOR antagonists. Selected clinical opioids and a naloxone positive control were tested as antagonists vs. 100 nM U50,488 at the KOR using ^35^S-GTPγS coupling. **A)** The data was normalized to the stimulation caused by 100 nM U50,488 (100%) or vehicle (0%) and reported as the mean ± SEM of N = 3 independent experiments. **B)** The resulting potency (IC_50_) and efficacy (I_MAX_) values are reported as the mean ± SEM. The I_MAX_ is normalized to the inhibition caused by the positive control naloxone (100%). Buprenorphine shows high efficacy and very high potency KOR antagonist activity, but the other clinical opioids show no evidence of antagonist activity.

At the KOR, buprenorphine showed a shift in binding affinity to potency of 27 nM to 1078 nM; however, the efficacy was very low (9.6%), consistent with buprenorphine’s known activity as a KOR antagonist [18]. Similarly, hydrocodone displayed no KOR binding, but moderate potency (236 nM) and low efficacy (9.8%) KOR activity, also suggesting potential antagonism. O-desmethyl-tramadol (5.9%), oxycodone (9.6%), and tapentadol (13.0%) also showed low potency and low efficacy KOR activity, potentially suggesting antagonist activity. Hydromorphone, morphine, and oxymorphone all displayed clear partial agonist activity, with low potencies favoring MOR selectivity. As some of these compounds suggested potential antagonist activity, we evaluated selected clinical opioids and a naloxone positive control in antagonist mode at the KOR (**Fig 3**). As expected, buprenorphine showed very high potency KOR antagonism, but hydrocodone, oxycodone, and tramadol displayed no KOR antagonist activity.

At the NOP, no clinical opioid produced any agonist activity, including buprenorphine. As buprenorphine is a low efficacy NOP partial agonist [34], our ^35^S-GTPγS assay may not have been sensitive enough to detect it. In CB1 binding from **Table 4**, only buprenorphine showed weak CB1 binding affinity. It was thus surprising to find in our activity assays (**Table 5**) that hydrocodone, hydromorphone, and morphine all showed low potency partial agonist activity at the CB1 (>41.5%). Even more surprising, buprenorphine showed low potency *inverse agonist* activity at the CB1. None of these associations has been reported in the literature.

For the monoamine transporter activity assays, O-desmethyl-tramadol, tapentadol, and tramadol all showed low potency but clear transport inhibition activity at the NET and SERT, as expected from the literature [20, 21]. Tapentadol and tramadol further showed the beginning of transport inhibition activity at very low potency at the DAT. Unexpectedly however, buprenorphine showed low potency transport *activation* activity at all 3 transporters (DAT > NET > SERT). The curves are incomplete, but buprenorphine shows good activity at the DAT, activating transport by 67.2%. This higher activity at the DAT may correlate with the observed low potency buprenorphine binding to the DAT (**Table 4**). All of the potential novel interactions detected during the binding and functional studies and not found in the literature are summarized in **Table 6**.

**Table 6:** Summary of novel drug and target interactions.

Potential New Findings	
Buprenorphine	CB1 inverse agonist; Monoamine transporter activator
Hydrocodone	KOR partial agonist; CB1 partial agonist; σ1R binding
Hydromorphone	CB1 partial agonist
Morphine	CB1 partial agonist
O-Desmethyl-Tramadol	DOR/KOR partial agonist
Oxycodone	None
Oxymorphone	DOR/KOR partial agonist
Tapentadol	KOR partial agonist; σ1R binding
Tramadol	None

### Behavioral testing of potential buprenorphine transporter activity

Some of the novel interactions summarized in **Table 6** are observed at low potencies, raising doubts as to whether they would be relevant at therapeutic doses. To test this question, we focused on the novel activating activity of buprenorphine at the monoamine transporters. We observed similar potencies at the transporters for tapentadol and tramadol, which are known to act at the transporters *in vivo* at therapeutic doses [20]. Also, *in vitro* transporter activity assays with these compounds show low potencies even with synaptosomal preparations from brain, potentially suggesting that *in vitro* transporter assays underestimate actual *in vivo* potencies [20]. We further reasoned that since monoamine transport inhibitors like amitriptyline are anti-nociceptive in chronic and neuropathic pain states, that the transport *activating* activity of buprenorphine should be pro-nociceptive [37].

**Fig 4:**
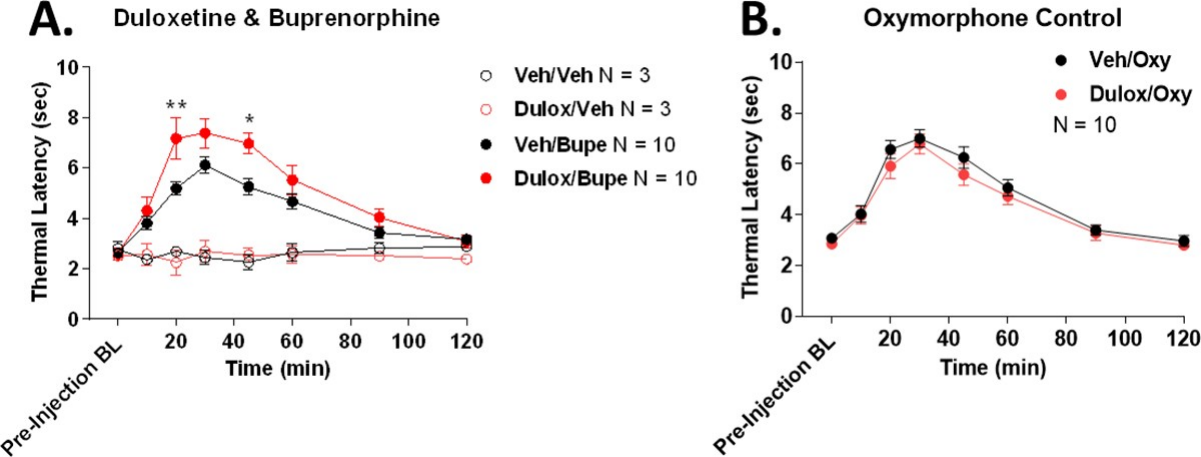
The monoamine transporter inhibitor duloxetine causes a buprenorphine-selective enhancement of tail flick anti-nociception. Male CD-1 mice were tested for tail flick anti-nociception baselines with 52°C water (10 second cutoff). The mice were then injected with duloxetine (20 mg/kg) or vehicle (1% Tween80 in saline) by the intraperitoneal route for 10 minutes, followed by subcutaneous buprenorphine (0.2 mg/kg), oxymorphone (0.3 mg/kg), or vehicle (saline). Tail flick latencies were then recorded over a 2 hour time course. Data reported as the latencies in raw seconds, mean ± SEM. Sample sizes of individual mice/group are noted in the graph legends. The Vehicle/Vehicle and Duloxetine/Vehicle groups were performed as one technical replicate. All other groups were performed as 2 technical replicates, with N = 5/group for each replicate. The same blinded experimenter performed all experiments. **A)** Vehicle or duloxetine alone had no effect on tail flick baselines. Duloxetine increased buprenorphine anti-nociception with an AUC increase of 50.6%. *, ** = *p* < 0.05, 0.01 vs. same time point Veh/Bupe group by 2 Way ANOVA with Fisher’s Least Significant Difference post-hoc test. **B)** Duloxetine had no effect on oxymorphone anti-nociception. *p* > 0.05.

We thus treated mice with the non-selective monoamine transport inhibitor duloxetine (20 mg/kg) 10 minutes prior to equi-efficacious ~A_50_ doses of buprenorphine (0.2 mg/kg) or oxymorphone (0.3 mg/kg). Oxymorphone was chosen as a comparison due to having the highest MOR selectivity along with no interactions outside of the opioid receptors (**Table 5**). Tail flick anti-nociception was measured as a behavioral output. We found that vehicle alone or duloxetine alone had no effect on tail flick baselines, but duloxetine treatment *increased* buprenorphine-mediated anti-nociception with an AUC increase of 50.6% (**Fig 4A**). By comparison, duloxetine had no effect on oxymorphone anti-nociception (**Fig 4B**). These results are consistent with duloxetine blocking the putative pro-nociceptive transport activating activity of buprenorphine, resulting in enhanced anti-nociception. The oxymorphone control suggests that this effect of duloxetine cannot be explained by MOR interaction alone. Overall these results provide support that even the low potency novel interactions found in this study (**Table 6**) could be endogenously relevant at therapeutic doses.

## Discussion

In this study, we comprehensively screened the molecular pharmacology of 9 clinically relevant opioids at 9 pain-related receptor targets (**Table 1**). Such a comprehensive screen has not been performed for these compounds to our knowledge, and our findings could potentially explain discrepancies between clinical opioids in their *in vitro* vs. *in vivo* τ values and lack of cross-tolerance (see Introduction). We found many expected interactions, including expected binding affinities and potencies at the MOR, and NET/SERT interaction for tapentadol and tramadol. These expected findings, rank order affinities/potencies, and the expected behavior of positive control compounds all validate our experimental approach [3].

This validation lends confidence to our unexpected findings, summarized in **Table 6**. Some of these findings are likely to be significant at therapeutic doses, such as σ1R binding that is only 2.3 fold over MOR affinity for hydrocodone and approximately equal affinity to MOR for tapentadol. Similarly, hydrocodone displays CB1 partial agonist activity that is only 3.1 fold over MOR potency, and O-desmethyl-tramadol displays DOR partial agonist activity that is equal in potency to the MOR. However even low potency interactions have the potential to be significant at therapeutic doses. We detected a highly novel but low potency transporter *activating* activity by buprenorphine; using the transporter inhibitor duloxetine, we show results consistent with a transport activation activity by buprenorphine at a moderate ~A_50_ dose in mice (**Fig 4**). These results suggest that even our low potency/affinity novel interactions should be explored for potential roles at therapeutic doses *in vivo*.

We did observe discrepancies in some cases between the binding (**Table 4**) and functional data (**Table 5**). While in some cases binding and function matched well—such as oxymorphone at the MOR (11 vs. 23.1 nM)—in others they did not, as multiple compounds at multiple targets showed functional activity without any apparent binding affinity (O-desmethyl-tramadol at DOR, multiple compounds at CB1 and NET, etc.). In a few cases, binding affinity was significantly greater than functional potency, such as buprenorphine at the DOR (34 nM affinity vs. 1684 nM potency). Since compounds like oxymorphone matched previously reported results well, and positive controls behaved as expected, the answer cannot be as simple as one assay being more or less sensitive than the other. Several potential explanations could be behind these discrepancies. For one, compounds could have high or low intrinsic efficacy, which can more or less efficiently translate binding into a functional response [12, 38]. By this explanation, O-desmethyl-tramadol could have high intrinsic efficacy at the DOR, while buprenorphine could have low intrinsic efficacy at the DOR. For another, some compounds could bind allosterically to produce a functional change, while radioligand binding will only measure displacement of orthosteric ligand binding [12]. This could most readily explain compounds that produced functional changes without detectable binding, such as O-desmethyl-tramadol which produced no binding displacement at the DOR up to a 10 μM concentration, but produced functional changes at the modest potency of 361 nM.

Related to these discrepancies, the affinity and potency values measured are in some cases lower than those reported elsewhere in the literature, particularly for hydrocodone (1757 nM affinity, 472 nM potency). However, our rank order of compounds matches well with the literature, along with the performance of our positive control compounds, suggesting the results are valid [3]. Contrary to common perception, reported molecular pharmacology values for opioid (and other) compounds can vary wildly, and are highly dependent on assay conditions, cell line, temperature used, and other variables. For example, in the case of fentanyl, an affinity range of 20,000 fold has been reported throughout the literature [3]. Considering all factors, our observations appear valid, within the range of literature values, and are confirmed by intrinsic factors such as positive control compounds and potency/affinity rank order.

Among our observed novel interactions, some may be significant, and fall into a few categories (**Table 6**). Hydrocodone, O-desmethyltramadol, oxymorphone, and tapentadol showed previously unreported KOR and/or DOR partial agonist activity. This is not surprising, as other opioids such as morphine have long been known to show KOR and DOR agonist activity [39, 40]. Evidence from MOR KO mice does suggest that morphine mediates anti-nociception fully through the MOR [4]; however, evidence with drugs like oxycodone suggests that the DOR and/or KOR could mediate part of the anti-nociceptive response with these drugs [19]. DOR and KOR activity should thus be explored as contributors to the pharmacological profile of hydrocodone, O-desmethyl-tramadol, oxymorphone, and tapentadol, especially as some of these compounds have very similar MOR and KOR/DOR potencies. KOR/DOR activity could be the mechanism behind lack of cross-tolerance between morphine and oxycodone [8], and could be involved with the above drugs.

We also found that hydrocodone, hydromorphone, and morphine have CB1 partial agonist activity, while buprenorphine has CB1 inverse agonist activity, all unreported; hydrocodone furthermore has a CB1 potency only 3.1 fold lower than MOR, suggesting potential activity at therapeutic doses. CB1 is an extremely widespread and highly expressed modulatory system in the brain, that among other roles, promotes anti-nociception by itself and in synergy with the opioid system [26, 41]. We hypothesize that if these drugs promote CB1-mediated anti-nociception, this effect could synergize with the opioid system and promote a dose-reduction effect to decrease opioid and CB1-mediated side effects, as has been shown with separate opioid and CB agonists [42]. In contrast, if buprenorphine acts as a CB1 inverse agonist, this could cause a pro-nociceptive effect, limiting potential anti-nociception (as we observed for buprenorphine transporter activity in **Fig 4**). These effects could again explain some discrepancies between opioids *in vitro* and *in vivo*, and could suggest drug discovery approaches to refine these CB1 vs. MOR activities to enhance anti-nociception.

Another novel finding is that hydrocodone and tapentadol bind to the σ1R with affinities quite close to that for the MOR (2.3 fold for hydrocodone, same for tapentadol), suggesting activity at therapeutic doses. The σ1R is an intracellular chaperone-like protein mainly expressed in the endoplasmic reticulum and strongly concentrated in the brain; it may act to selectively amplify signal transduction, and σ1R antagonists are being explored as novel analgesics [27, 43, 44]. Hydrocodone and tapentadol binding to the σ1R could thus promote anti-nociception. However one drawback to our approach is that the binding assay does not reveal the functional impact of these drugs on the σ1R. Further research will thus be required to determine how hydrocodone and tapentadol modulates σ1R function, and what is the resulting impact on anti-nociception.

Lastly, we found that buprenorphine has a monoamine transporter activating activity, a highly novel finding. Although low potency, our *in vivo* experiments suggest that this activity could be relevant at moderate doses (**Fig 4**). In the entire clinical pharmacopeia, we could find no other drugs that activate monoamine transport. In the entire literature, we could find only a single report of 2 natural product compounds that causes monoamine transporter activation [45]. The literature and our data (**Fig 4**) suggests that this effect is pro-nociceptive. However, as we see the highest efficacy at the DAT, this effect could act to reduce dopamine release in the brain, which we would expect to reduce reward and drug abuse liability [46]. This effect could thus explain the efficacy of buprenorphine as a treatment for drug addiction, and could be further enhanced to improve efficacy for this indication [47].

These findings should also be placed into a broader context of novel drug discovery and development for the opioid receptors. New findings in regards to the molecular pharmacology of ligand:receptor interaction and downstream signaling pathways suggest additional complexity to the system that can be utilized to improve opioid therapy, in greater detail than the simple molecular pharmacology screening performed in this study (we review some of these novel approaches in [48]). For instance, receptors can interact with each other in homodimer or heterodimer pairs, or higher order oligomeric complexes, that evoke unique signaling when compared to the monomers that can be taken advantage of to improve opioid therapy [49]. We recently created a bivalent antagonist specific for the mu-delta opioid receptor heterodimer that enhances opioid anti-nociception while decreasing side effects like withdrawal, suggesting that heterodimers could be promising future drug discovery targets [32]. Looking to downstream signaling, seminal work from the lab of Laura Bohn identified the signaling regulator βarrestin2 as a molecule which decreased morphine anti-nociception while enhancing side effects [50–52]. This finding led to the development of ligands biased *against* βarrestin2 recruitment, which showed decreased side effects in animal models, and some of which are in clinical trials [53–55]. These novel approaches show that the simple ligand:receptor interactions profiled in this manuscript are only part of the story, and that the clinical opioids profiled here among other novel opioid drugs should be examined in a broader context of receptor interaction which could include pathway bias, preference for heterodimer interaction, or similar. A full understanding of these factors will likely be necessary for the successful creation of new opioid drugs without the side effects of current ligands.

## Supporting information

S1 File Supporting info—All raw data.7z. (7Z)

## Abbreviations

(AUC): Area Under the Curve
(K_D_) and competition (K_I_): Binding constant–saturation
(CB1): Cannabinoid Receptor Type 1
(CHO): Chinese Hamster Ovary
(E/I_MAX_): Efficacy
(FBS): Fetal Bovine Serum
(GPCR): G Protein Coupled Receptor
(GDP): Guanosine Diphosphate
(HA): Hemagglutinin
(HEK293, HEK): Human Embryonic Kidney
(KO): Knockout
(B_MAX_): Maximum Number of Binding Sites
(MOR/DOR/KOR): Mu/Delta/Kappa Opioid Receptor
(NOP): Nociceptin Receptor
(NET/SERT/DAT): Norepinephrine/Serotonin/Dopamine Transporter
(P/S): Penicillin/Streptomycin
(EC/IC_50_): Potency
(σ1R): Sigma-1 Receptor
